# TARGETING SOCIALLY ISOLATED OLDER ADULTS: THE INTERNET-BASED CONVERSATIONAL ENGAGEMENT TRIAL (I-CONECT)

**DOI:** 10.1093/geroni/igae098.3730

**Published:** 2024-12-31

**Authors:** Hiroko Dodge, Chao-Yi Wu, Liu Chen, Kexin Yu

**Affiliations:** Harvard Medical School, Massachusetts General Hospidal, Framingham, Massachusetts, United States; Massachusetts General Hospital, Harvard Medical School, Massachusetts General Hospital, Harvard Medical School, Massachusetts, United States; Massachusetts general hospital, Boston, Massachusetts, United States; Oregon Health and Science University, Portland, Oregon, United States

## Abstract

Social isolation is a risk factor for dementia. In the recently completed randomized controlled trial named I-CONECT (www.i-conect.org; ClinicalTrials.gov: NCT02871921), we investigated the impact of frequent social interactions via webcam/internet on the cognitive function and emotional well-being, recruiting socially isolated older adults aged 75 and above. Participants in the experimental group engaged in semi-structured conversations with interviewers, prompted by daily themes and associated pictures, 4 times per week (30 minutes/session) for 6 months. The control group received only brief weekly phone check-ins. A total of 186 subjects (86 with normal cognition, 100 with mild cognitive impairment [MCI]) were randomized. We earlier reported that global cognitive function (the primary outcome), as measured by the Montreal Cognitive Assessment (MoCA), improved by nearly 2 points in the MCI experimental group compared to the control group at 6 months (effect size: Cohen’s d = 0.73) (Dodge et al., 2024, doi: 10.1093/geront/gnad147). Functional MRI data suggested a trend toward increased connectivity in the dorsal attention network favoring the experimental group. Social satisfaction improved in both groups. Our new results not reported in the topline results paper include: (1) Participants in the experimental group increased the frequency of their social interactions over time compared to the control group, and (2) Individual Treatment Response analysis showed that participants in the top 30% of responders could delay cognitive decline by 6 months or more. We will summarize all findings from this trial thus far and discuss future directions.

